# Influence of Implant Macrogeometry on Primary Stability in Different Polyurethane Bone-Density Models: An In Vitro Study

**DOI:** 10.3390/bioengineering13101162

**Published:** 2026-10-04

**Authors:** Margherita Tumedei, Ugo Graziani, Alessandro Cipollina, Renato Ori, Adriano Piattelli, Ugo Covani, Lorenzo Enia, Calogero Pitruzzella, Francesca Mangione

**Affiliations:** 1 Department of Biomedical, Surgical and Dental Sciences, University of Milan, 20122 Milan, Italy; 2 Private Practice, 00184 Rome, Italy; ugograziani@gmail.com (U.G.); studio.renatoori@gmail.com (R.O.); 3 Private Practice, 92019 Sciacca, Italy; alexandros1960@libero.it; 4 Saint Camillus International University of Health Sciences (UniCamillus), 00131 Rome, Italycovani@covani.it (U.C.); 5 Istituto Stomatologico Toscano (IST), 55049 Viareggio, Italy; 6 Private Practice, 90047 Partinico, Italy; cmo_enialorenzo@hotmail.com; 7 Private Practice, 92026 Favara, Italy; pitruzzella.calogero@libero.it; 8 Unité Mixte de Recherche (UMR-S 1333) Oral Health, Université Paris Cité, Sorbonne Paris Nord, Institut National de la Santé et de la Recherche Médicale (INSERM), 92120 Montrouge, France; francesca.mangione@parisdescartes.fr; 9 Department of Odontology, Assistance Publique–Hôpitaux de Paris (AP-HP), Henri Mondor University Hospital, 94000 Créteil, France

**Keywords:** dental implants, primary stability, thread geometry, resonance frequency analysis, polyurethane foam, bone density

## Abstract

Background: Primary stability (PS) is a crucial determinant of long-term dental implant success, particularly in compromised or low-density bone sites. While macrogeometry is known to influence initial mechanical anchorage, the specific contribution of thread dimensions across varying bone densities remains incompletely understood. This in vitro study evaluated the influence of two different implant macrogeometries characterized by distinct thread configurations on primary implant stability within standardized polyurethane foam models representing different bone qualities. Methods: Two implant designs featuring distinct thread profiles were tested: the Evolution KRI (larger threads, pitch 1.8–2.2 mm, depth 0.55–1.025 mm) and the Evolution PRI (smaller threads, pitch 1.0 mm, depth 0.5 mm), across three fixture diameters (4.0, 4.5, and 5.0 mm). Implants were inserted into three standardized polyurethane block configurations: Group I (25 PCF uniform block, simulating D2 dense trabecular bone), Group II (10 PCF trabecular base with a 3 mm 40 PCF cortical layer, simulating D3 bone), and Group III (12.5 PCF trabecular base with a 2 mm 40 PCF cortical layer, simulating D4 bone). Each experimental group included 10 implants (n = 10). Two RFA measurements were collected for each implant and averaged prior to statistical analysis. Group comparisons were performed using one-way ANOVA followed by Tukey’s post hoc test. Primary stability was quantitatively assessed using Resonance Frequency Analysis (RFA) to record Implant Stability Quotient (ISQ) scores. Results: Statistically significant differences in ISQ values were observed among the experimental groups ($F=9.909,p<0.0001,R^{2}=0.4785$). Implants featuring larger threads (KRI) demonstrated consistently higher mean ISQ scores compared to smaller-thread implants (PRI) across all bone densities. In Group I (D2 model), KRI implants achieved a significantly higher mean ISQ than PRI implants (67.1 ± 1.91 vs. 65.1 ± 1.43; *p* = 0.0472). In Group II (D3 model with 3 mm cortical layer), this difference was most pronounced, with KRI reaching 68.5±0.58 compared to 64.8 ± 2.15 for PRI (*p* < 0.0001). In Group III (D4 model with 2 mm cortical layer), KRI maintained a higher stability trend (68.0±0.93 vs. 66.5±1.42), though the inter-system difference did not reach statistical significance (p=0.2401). Inter-group comparisons confirmed that the highest overall primary stability was obtained by large-thread implants in the presence of a 3 mm cortical layer. Conclusions: Thread macrogeometry significantly influences primary implant stability; the tested KRI macrogeometry demonstrated higher ISQ values than the PRI macrogeometry under specific polyurethane configurations, particularly in intermediate trabecular–cortical bone structures.

## 1. Introduction

Bone density at the level of the implant insertion bone sites is one of the most important parameters influencing primary stability (PS) [1,2]. PS can be defined as the lack of implant micromotion soon following implant placement [3].

Higher PS was noticed in compact bone when compared with medullary bone tissue [4,5]. Low PS has been reported to have a higher risk of implant failure or implant loss, while, on the other hand, high PS produces the most favorable situations for implant integration [3]. This connection of PS with bone insertion sites density has a paramount meaning in implant-prosthodontic rehabilitations, implant loading, and implant long-term success [1,3]. PS indicates the initial biomechanical connection between implant and host bone [6]. PS is adversely influenced by the presence of low density and low quality host bone [6]. Also, the implant profile and macrogeometry may significantly affect the extent and measure of PS [1]. Falco et al., Romanos et al., Mohlenrich et al. and Gehrke et al. reported that bone density, implant shape, implant design, implant surface characteristics, compressive stresses at the interface, the bone-to-implant percentage (BIC), the accurate fit in the bone and surgical technique play a key role in obtaining a good PS [3,4,7]. insertion torque (IT) and removal torque (RTV) provide, for the most part, equal and similar estimates, while Resonance Frequency Analysis (RFA) with the Implant Stability Quotient (ISQ) values conveys different results, concerning the interaction, at the interface, between the implant and the host bone site [1]. IT assesses the compression produced by the implant placement into the surgical site [5]. In general, IT determines the PS, which is considered the most relevant element in effective implant treatment [2]. IT is enhanced in accordance with the existence, thickness and density of a cortical layer [2]. Positive correlations were found between IT and RFA, especially in the posterior maxilla, whereas no correlations between RFA, IT and bone density using histomorphometric data were found [2,6]. In Type I Bone, the IT was described to be notably and constantly enhanced in contrast to Type IV Bone [5]. Implant diameter seems to have a superior impact on PS than implant length [3,7].

The features of polyurethane may make it a first-rate synthetic material for implant testing and analysis [1], due to their biomechanical characteristics similar to those of human bone. Various papers use different classifications of the polyurethane density: Type I Bone (40 per cubic feet/0.64 g/cm^3^), Type IV Bone (15 per cubic feet/0.24 g/cm^3^), D1 0.48 g/cm^3^, D2 0.32 g/cm^3^, D3 0.11 g/cm^3^, D4 0.08 g/cm^3^ [3]. Polyurethane foam blocks and sheets have been standardized as a material for the tests of orthopedic devices, instruments and also of dental implants [2,8]. The physical characteristics of polyurethane are homogenous all over their mass, so as to gain a good standardization of the processes and avoid inserting potential origins of bias in the calculations such as those that could occur in biological specimens [2,4]. In the past few years, several papers, from the Laboratory of some of the present Authors, on the use of polyurethane foam to test several different features of dental implants have been published [9,10].

Although several studies have demonstrated that implant macrogeometry may influence primary stability, limited evidence is available regarding the comparative behavior of different commercially available implant macrogeometries under standardized polyurethane models reproducing different bone-density conditions [3] The aim of the present study was to compare two implant systems characterized by different macrogeometries and thread configurations in three standardized polyurethane bone models.

## 2. Materials and Methods

### 2.1. Implants

#### 2.1.1. PRI

The Evolution PRI implant is specifically engineered to obtain an optimal primary stability in high-density cortical bone types D1 and D2. The geometry is designed to obtain mechanical performance and precise cutting capability with 0.5 mm thread depth and 1.00 mm pitch. The implant is characterized by active thread cuts capable to cut dense cortical bone without generating over-friction or thermal stress on adjacent tissue (Table 1).

#### 2.1.2. Evolution KRI

The Evolution KRI implant system is engineered to achieve optimal primary stability in low-density medullary bone D3 and D4 bone types. Each individual implant diameter is characterized by a specific thread design to maximize cutting efficiency and d primary retention in soft or cancellous bone structures (Table 1).

The 4.0 mm diameter implant presents a 0.55 mm thread depth with a 1.80 mm pitch.

This differential thread profile is designed to produce a condensation of surrounding cancellous bone during insertion, resulting in an efficient primary mechanical anchorage in low-density bone.

### 2.2. Study Design

**Figure 1:**
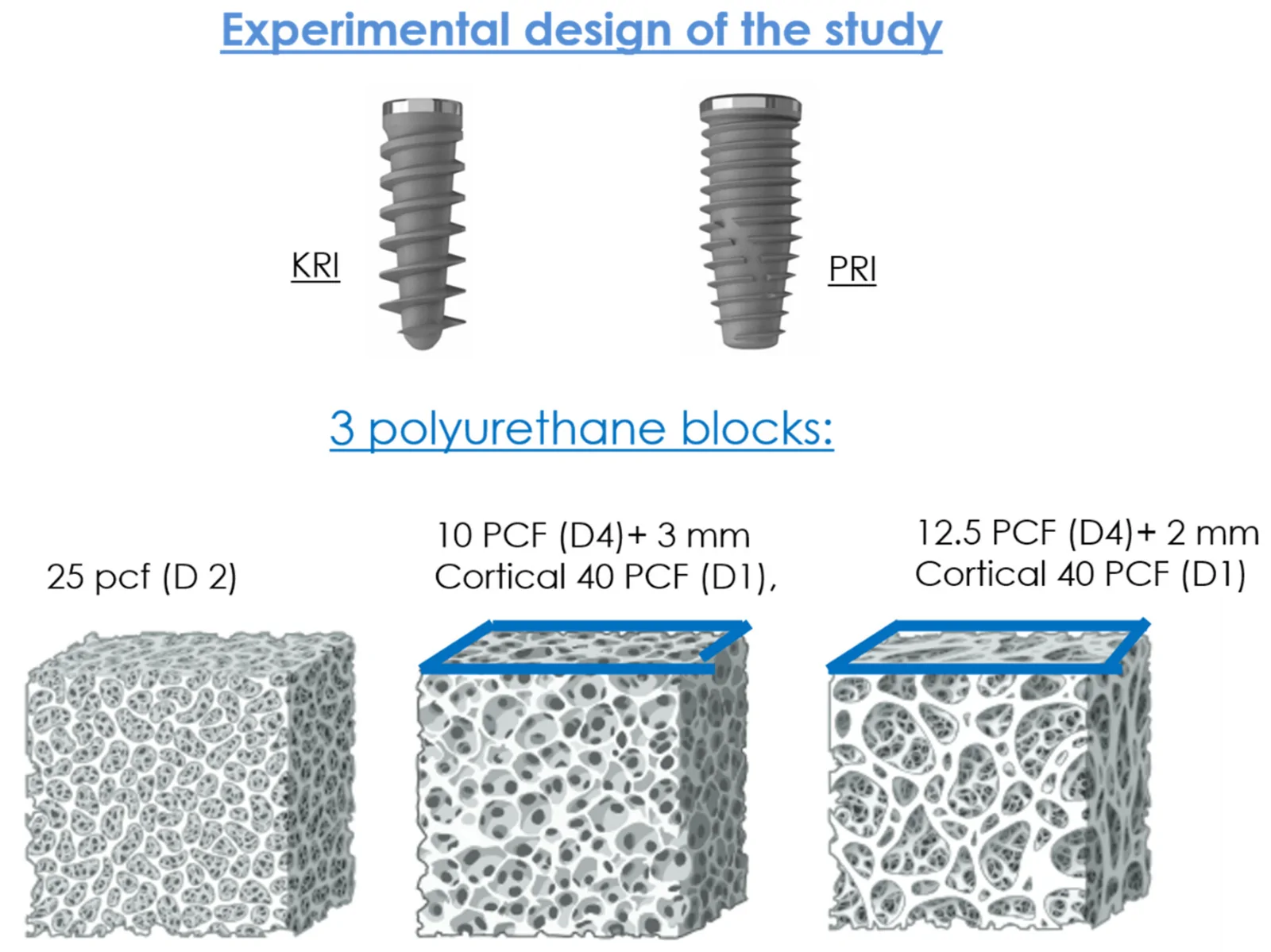
Experimental design of the study. Schematic representation of the implant systems evaluated—Evolution KRI (larger threads, wide coil, tapered core) and Evolution PRI (smaller threads, compact core)—and the three polyurethane block configurations used to simulate different bone densities: Group I (25 PCF, D2 bone simulation), Group II (10 PCF trabecular base + 3 mm 40 PCF cortical layer, D3 bone simulation), and Group III (12.5 PCF trabecular base + 2 mm 40 PCF cortical layer, D4 bone simulation).

Three types of polyurethane blocks have been tested to simulate various clinical conditions: 25 PCF (dense bone simulation), composite 10 PCF + 3 mm Cortical (40 PCF), and composite 12.5 PCF + 2 mm Cortical (40 PCF). Only one implant diameter per implant system was investigated. Therefore, implant diameter was not included as an independent experimental factor in the statistical analysis. Each experimental group consisted of n = 10 implants (Figure 1).

### 2.3. Primary Stability Measurement ISQ

The implants were inserted using a special type of device, a brushless motor, BMS Implant I.S.Q.A. (Implant Stability Quotient Analyzer), (Esacrom, Imola, Bologna, Italy). The Resonance Frequency Analysis (RFA) was, then, assessed by an electronic device (Osstel, Columbia, MD, USA) after the screw positioning. The values were registered in accordance to the Implant Stability Quotient (ISQ) score. The RFA assessment was performed two times for each implant. Two RFA measurements were obtained for each implant, and their mean value was used for statistical analysis. Repeated measurements were not treated as independent observations.

All implant insertions were performed by a single experienced operator using standardized drilling and insertion protocols. The insertion procedure was conducted using the same motor settings for all specimens.

### 2.4. Statistical Analysis

The study data were registered and analyzed by a special data form produced by the statistical software package GraphPad 6 (Prism, San Diego, CA, USA). The normality distribution of the study data was evaluated by the D’Agostino and Pearson test. The study variables’ significance between the study groups was evaluated by the one-way ANOVA test followed by the Tukey post hoc test. The level of significance was set at *p* < 0.05.

## 3. Results

### 3.1. Descriptive Analysis of ISQ

The analysis of central tendency and dispersion parameters highlights a distinct variability in ISQ values across the different configurations. In Group I, KRI implants demonstrated a higher mean stability value of 67.1 ± 1.91 compared to the PRI implants at 65.1 ± 1.43 (Table 2). This divergence becomes most pronounced in Group II, where the KRI system reaches a mean stability of 68.5 ± 0.58, while the PRI system settles at 64.8 ± 2.15. In Group III, the means are closer and were KRI: 68.0 ± 0.93 and PRI: 66.5 ± 1.42, although the KRI system consistently maintains a higher stability trend.

### 3.2. Multiple Comparisons

**Figure 2:**
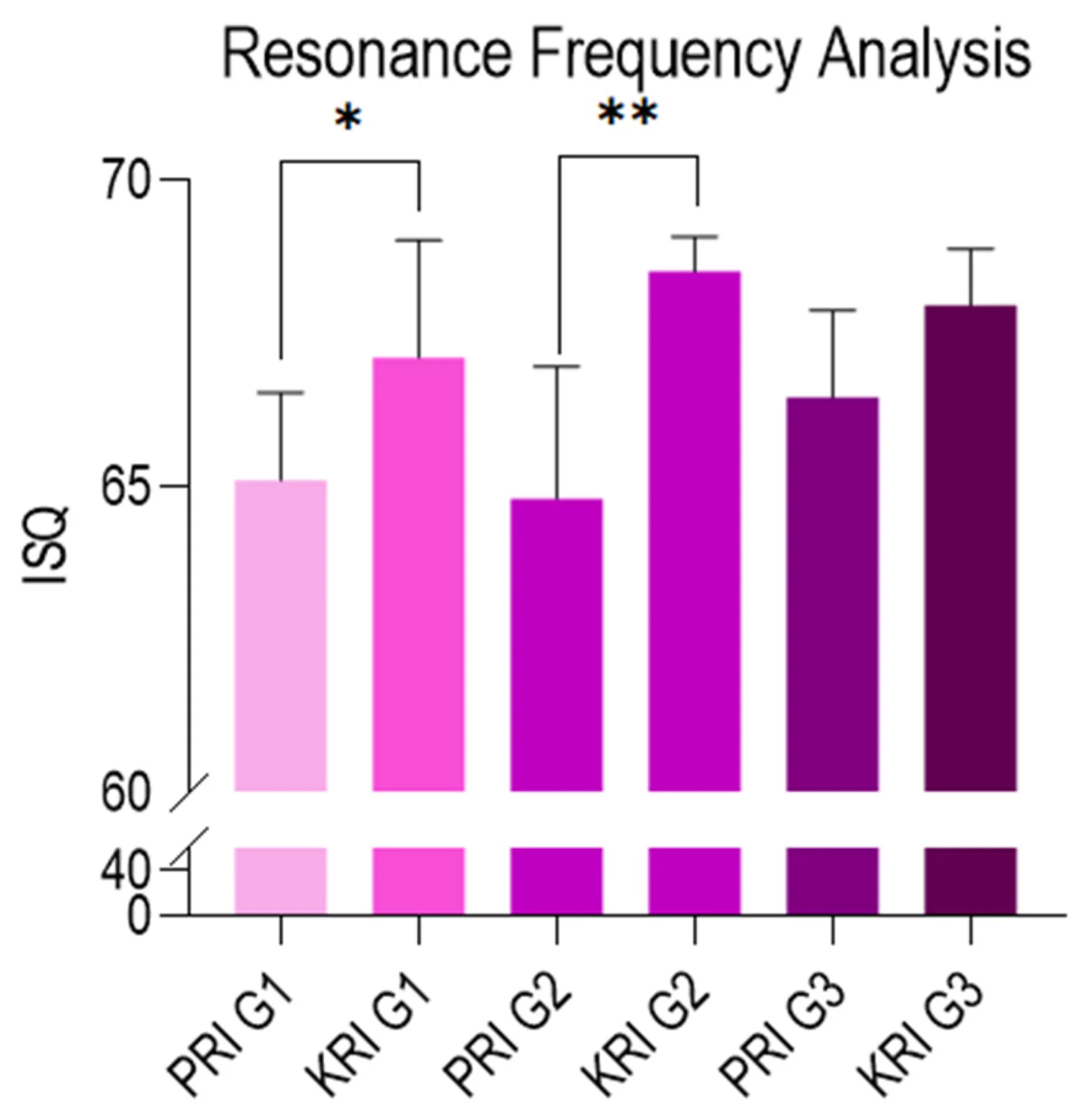
Resonance Frequency Analysis (RFA) results. Bar plot depicting mean Implant Stability Quotient (ISQ) values and standard deviations across experimental groups for PRI and KRI implants in different bone-density models [* p < 0.05; ** p < 0.001; *** p < 0.0001; **** p > 0.00001].

The one-way ANOVA model compared the six predefined experimental groups generated by the combination of implant system (PRI/KRI) and polyurethane configuration (Groups I–III) (Table 3 and Table 4; Figure 2). Significant differences among group means were identified (*F* = 9.909; *p* < 0.0001). The R-squared value (R^2^ = 0.4785) indicates that the model explains approximately 47.8% of the variance observed in the ISQ values. Regarding homoscedasticity, the results were varied: while the Brown–Forsythe test showed no significant differences in standard deviations (*p* = 0.0856), Bartlett’s test highlighted an inhomogeneity of variance (*p* = 0.0061), suggesting that the data distribution across subgroups is not perfectly uniform. Tukey’s multiple comparison test identifies where the most relevant clinical discrepancies between the implant systems lie. In terms of intra-system comparisons of PRI vs. KRI, the KRI system proved to be significantly more stable than the PRI in Group 1 (Diff: −2.0; *p* = 0.0472). In Group 2, this difference in favor of the KRI was highly significant (Diff: −3.7; *p* < 0.0001), whereas in Group 3, the difference between PRI and KRI did not reach statistical significance (*p* = 0.2401). When examining cross-comparisons between groups, the KRI G2 implant exhibited significantly superior stability performance compared to all PRI configurations (G1, G2, and G3). Furthermore, no significant differences were detected between the various groups of the same PRI implant (e.g., PRI G1 vs. PRI G2, *p* = 0.9976), indicating a consistent behavior of the material regardless of the specific test group conditions.

## 4. Discussion

The present study evaluated only RFA-derived primary stability under controlled synthetic conditions. No conclusions can be drawn regarding osseointegration, secondary stability, marginal bone maintenance, implant survival, or clinical success.

A major limitation of the present study is that the two implant systems differed in several macrogeometric characteristics beyond thread dimensions, including pitch, core configuration, and implant body design. Consequently, the independent contribution of thread depth or pitch cannot be isolated, and the results should be interpreted as a comparison between two implant macrogeometries rather than an evaluation of a single thread-design parameter.

A good initial PS during implant insertion is of fundamental importance for a long-term successful implant treatment [11].

This biomechanical coupling between implant and receiving host bone is of paramount value and represents a compacting biomechanical fixation that plays a key role to avoid deleterious micromotion at the interface [12]. PS is even more important in low-density and low-quality bone, when a decreased host bone may lead to an increased possible risk of micromovements at the implant–bone interface [13].

A systematic review with meta-analysis evaluated different implant site preparations that could enhance PS in soft bone. Notable results were reported when comparing osseodensification, underdrilling and the osteotome technique to conventional drilling [14].

Two of the most essential aspects producing an elevated level of PS after implant placement are implant macrogeometry and thread profile [15]. These implant characteristics are particularly relevant in low-quality and low-density bone, and an high PS in these anatomical regions is pivotal for the long-term implant success [16].

In a study using polyurethane foam in bone Types 3 and 4, it has been reported that implant macrogeometry and bone density had the most decisive impact on PS [17].

In the present study, cortical bone seems to have an extremely relevant biomechanical role. These data are supported by research that found that the presence or the addition of a thick cortical bone improved PS, with higher IT and ISQ values [18]. These results were, furthermore, confirmed in a radiological and clinical study on elderly patients, evaluating the mandibular cortical index (MCI) and ISQ. A significant decrease of the ISQ values was observed in patients presenting an important bone demineralization. The PS could, then, be reduced in patients with spongy mandibles [11].

In the present in vitro, polyurethane foam study, the large-thread implants constantly demonstrated higher ISQ values when compared to the small-thread implants, across all the different polyurethane densities. The present findings are consistent with previous polyurethane studies demonstrating that implant macrogeometry may significantly influence primary stability under standardized conditions. Similar observations showed that the effect of implant design depends not only on implant geometry but also on the density and structural characteristics of the surrounding substrate. Nevertheless, recent clinical investigations and systematic reviews have indicated that increases in insertion torque or ISQ values do not necessarily translate into clinically relevant improvements in implant survival or marginal bone preservation. Consequently, the present findings should be interpreted as biomechanical observations rather than evidence of clinical superiority. Hernandez, in a recent systematic review with the use of 25 studies, found that wide-thread dental implants improved PS and marginal bone levels (MBLs) in immediate implant insertion protocols, particularly in soft bone. This Author reported that increased thread depth, wider or more aggressive threads, and tapered implant bodies mostly enhanced IT and ISQ numbers remarkably in D3–D4 bone and post-extraction sockets. Wide-thread implant macrogeometry seemed to augment PS in immediate implant insertion with positive results both for implant long-term survival and success and for good marginal bone outcomes [19]. It should be noted that Groups II and III differed not only in cortical thickness but also in trabecular density. Therefore, the observed differences cannot be attributed exclusively to cortical thickness, and the relative contribution of each variable remains undetermined.

As has already been reported, dental implant PS can be analyzed by IT and RFA. Lages et al. did a systematic review, using 12 studies, evaluating the possible relationship between these two values, and they concluded that no statistically significant correlation between ISQ and IT was demonstrated and that these two values were autonomous and not equivalent approaches to evaluate PS [20]. On the other hand, another study showed that ISQ had a mild connection with IT, bolstering its function as a supplementary gauge of PS. Available research does not sustain ISQ values as autonomous indicators of marginal bone levels or implant survival [21].

Undersized osteotomy and osseodensification represent approaches in the preparation of host bone sites to augment PS using separate biomechanical operating principles. In an in vitro study using Type IV polyurethane blocks with a dense cortical layer, it was reported that undersized osteotomy increased IT, due to a greater rotary resistance during implant placement, while, on the other hand, osseodensification augmented the ISQ, probably for a greater bone rigidity [13].

Even if it has been reported that PS may determine a better secondary stability, a high PS could produce adverse effects on the marginal bone level [22].

Marginal bone level is very important for the long-term implant success. Fu et al. performed a clinical study on 90 patients, correlating IT and bone density and ISQ and implant diameter. Bone density had a relevant influence on IT values and on MBL. Implant diameter had a more relevant effect when compared with implant length on both IT and ISQ. Moreover, bone density had a key role in IT and ISQ values. Bone density and IT here were more important than ISQ on MBL [23]. Also, Popovski et al. reported that implant length did not significantly affect this stability [24].

In a histological and histomorphometrical analysis of huma failed implants, it was reported that implants with an ISQ lower than 40 were irretrievably lost, without any possibility to save them. In fact, statistically significant correlations were observed between an ISQ lower than 36 and irrevocably failed implants [25].

The fact, however, that the present data were not uniform in all the experimental groups could point to the fact that the results were related not only to the implant macrostructure, but also by the structure of the surrounding material. The highest PS values were observed in the 10 PCF + 3 mm cortical model, corresponding to a D3 bone structure, further confirming the extreme importance of the presence of a cortical layer. It must, moreover, be underlined that the highest polyurethane density (25 PCF), in higher bone densities did not produce the highest ISQ values. Such data point to the fact that PS cannot be explained only in relationship with trabecular bone density. Both implant systems presented a great level of biomechanical interaction. The absence of statistically significant differences between the two implant systems in the D4 bone type (12.5 PCF + 2 mm cortical configuration) furthermore could point to the fact that only the implant macrostructure cannot completely get over the biomechanical constraints dictated by the adjacent bone structure. A relevant aspect of the present study was the addition of a dense cortical layer. From a biomechanical point of view, great importance seems to be related to the presence of a cortical layer. Polyurethane foam has repeatedly demonstrated the ability to allow consistent, standardized, homogenous, and reproducible results to be obtained. These features of this material minimize the biological variability found in human bone, allowing evaluations in controlled biomechanical environments. These in vitro studies have, naturally, some shortcomings due to the fact that a synthetic polymer cannot replicate bone remodeling, vascularization, osseointegration, and the response of vital bone to loading. Overall, the present findings indicate that both implant macrogeometry and bone characteristics play a key role in implant PS. The better PS results were obtained with the use of a larger threads, but it must also be underlined the relevant importance of the receiving bone site structure.

The biomechanical advantage that could be attributed to a particular implant design seems to be greatly contingent also on cortical and trabecular bone structure. Additional studies are needed to precisely correlate these findings with their clinical relevance under more biologically-oriented scenarios.

## 5. Conclusions

Within the limitations of this in vitro polyurethane model, the KRI implant macrogeometry showed higher ISQ values than the PRI macrogeometry in Groups I and II, whereas no statistically significant differences were observed in Group III. These results imply that PS appears to be affected not only by the implant macrogeometry but also by the features of the receiving host bone. The biomechanical advantage associated with a specific implant design appears to be highly dependent on trabecular–cortical architecture. Further studies are required to determine the clinical relevance of these findings under more biologically characteristic scenarios. Further biomechanical and prospective clinical studies are needed to determine whether these differences translate into clinically relevant outcomes.

## Figures and Tables

**Table 1:** Technical and clinical characteristics of the evaluated implant systems. Detailed comparison of macrogeometric parameters, thread designs, placement depths, and clinical indications for Evolution KRI and Evolution PRI implants.

Characteristics	Implant KRI	Implant PRI
Geometry	Conical, wide coil, tapered core	Core represented (higher pressure)
Threads Pitch	2 mm	1 mm
Joint	Hexagonal	Hexagonal
Clinical indication	Bone D2–D3–D4	Compact bone/increased stability
Positioning	Crestal level	Crestal level
Test diameters	4.0	4.0

**Table 2:** Descriptive statistics of PRI and KRI implants performance in group I, II and III.

	GROUP I		GROUP II		GROUP III	
	PRI	KRI	PRI	KRI	PRI	KRI
25% Percentile	63.8	65.3	63.8	68	65.4	67
Median	65.3	67.5	65.5	68.5	67	67.8
75% Percentile	66	69	66.1	69	67.5	69
Mean	65.1	67.1	64.8	68.5	66.5	68
Std. Deviation	1.43	1.91	2.15	0.577	1.42	0.926
Std. Error of Mean	0.452	0.605	0.68	0.183	0.45	0.293
Lower 95% CI of Mean	64.1	65.7	63.3	68.1	65.4	67.3
Upper 95% CI of Mean	66.1	68.5	66.3	68.9	67.5	68.6

**Table 3:** One-way analysis of variance (ANOVA) and homoscedasticity tests summary. Statistical summary of ISQ differences across experimental groups (F-value, p-value, $R^{2}$), along with Brown–Forsythe and Bartlett’s variance homogeneity assessments. [** p < 0.001].

ANOVA Summary	
F	9.909
*p* value	<0.0001
*p* value summary	
R square	0.4785
Brown–Forsythe test	
F (DFn, DFd)	2.054 (5, 54)
*p* value	0.0856
*p* value summary	ns
Bartlett’s test	
Bartlett’s statistic (corrected)	16.27
*p* value	0.0061
*p* value summary	**

**Table 4:** Tukey’s post hoc multiple comparisons test. Post hoc pair-wise statistical analysis comparing ISQ values intra-system and inter-group, including mean differences, 95% confidence intervals, adjusted p-values, and significance levels [* p < 0.05; ** p < 0.001; *** p < 0.0001].

Tukey’s Multiple Comparisons Test	Mean Diff,	95.00% CI of Diff,	Significant?	Summary	Adjusted *p* Value	
PRI G1 vs. KRI G1	−2	−3.985 to −0.01522	Yes	*	0.0472	A–B
PRI G1 vs. PRI G2	0.3	−1.685 to 2.285	No	ns	0.9976	A–C
PRI G1 vs. KRI G2	−3.4	−5.385 to −1.415	Yes	***	<0.0001	A–D
PRI G1 vs. PRI G3	−1.35	−3.335 to 0.6348	No	ns	0.3506	A–E
PRI G1 vs. KRI G3	−2.85	−4.835 to −0.8652	Yes	**	0.0012	A–F
KRI G1 vs. PRI G2	2.3	0.3152 to 4.285	Yes	*	0.0143	B–C
KRI G1 vs. KRI G2	−1.4	−3.385 to 0.5848	No	ns	0.3109	B–D
KRI G1 vs. PRI G3	0.65	−1.335 to 2.635	No	ns	0.9262	B–E
KRI G1 vs. KRI G3	−0.85	−2.835 to 1.135	No	ns	0.8022	B–F
PRI G2 vs. KRI G2	−3.7	−5.685 to −1.715	Yes	***	<0.0001	C–D
PRI G2 vs. PRI G3	−1.65	−3.635 to 0.3348	No	ns	0.1558	C–E
PRI G2 vs. KRI G3	−3.15	−5.135 to −1.165	Yes	***	0.0003	C–F
KRI G2 vs. PRI G3	2.05	0.06522 to 4.035	Yes	*	0.0391	D–E
KRI G2 vs. KRI G3	0.55	−1.435 to 2.535	No	ns	0.9628	D–F
PRI G3 vs. KRI G3	−1.5	−3.485 to 0.4848	No	ns	0.2401	E–F

## Data Availability

All experimental data to support the findings of this study are available upon request after contacting the corresponding author. The authors have annotated the entire data building process and empirical techniques presented in the paper.
